# Koshihikari: a premium short-grain rice cultivar – its expansion and breeding in Japan

**DOI:** 10.1186/s12284-018-0207-4

**Published:** 2018-04-09

**Authors:** Asako Kobayashi, Kiyosumi Hori, Toshio Yamamoto, Masahiro Yano

**Affiliations:** 1 Fukui Agricultural Experiment Station, 52-21 Ryomachi, Fukui, 918-8215 Japan; 2 National Agriculture and Food Research Organization (NARO), Institute of Crop Science, 2-1-2, Kannondai, Tsukuba, Ibaraki, 305-8518 Japan

**Keywords:** Eating quality, Koshihikari, Marker-assisted breeding, Quantitative trait locus, Temperate *japonica* group

## Abstract

Koshihikari, a Japanese short-grain rice cultivar, was developed in 1956, more than 60 years ago. Despite its age, it has been the most widely grown cultivar in Japan for more than 35 years, making it the most important rice for the Japanese people. In its early days, there was no reason to predict that Koshihikari would become so widely disseminated. However, since the end of the post–World War II food shortage in the 1960s, Japanese preferences changed from high productivity to good eating quality. This triggered wide expansion of Koshihikari cultivation due to the cultivar’s excellent taste and texture. With increasing cultivation of Koshihikari in Japan, several good agronomic characteristics beyond its high eating quality became apparent, such as its good adaptation to different environments, tolerance to pre-harvest sprouting, and cold tolerance during the booting stage. These characteristics outweigh drawbacks such as its low blast resistance and low lodging resistance. The popularity of Koshihikari influenced subsequent rice breeding trends at regional agricultural experimental stations, and the characteristics of newly developed rice cultivars in Japan are usually rated relative to Koshihikari, which is used as the benchmark. Koshihikari was the first *japonica* rice cultivar whose whole genome has been sequenced by means of next-generation sequencing. Furthermore, comparison of the genomes of Koshihikari and Nipponbare has provided detailed insights into the genetic diversity of Japanese rice cultivars relative to that in rice populations elsewhere in the world. Further progress in rice genomics is gradually unlocking the mechanisms that underlie the agronomic characteristics of Koshihikari. To support both research and the development of novel rice cultivars, a series of isogenic and near-isogenic lines in the Koshihikari genetic background have been continuously developed. These new findings and materials will facilitate genomics-assisted rice breeding, eventually leading to superior cultivars.

**Electronic supplementary material:**

The online version of this article (10.1186/s12284-018-0207-4) contains supplementary material, which is available to authorized users.

## Background

Rice (*Oryza sativa* L.) is a staple food in Japan. It was introduced into Japan from China’s Yangtze river basin and downstream catchments about 2000 to 3000 years ago, and it has been cultivated throughout the country ever since (reviewed by Matsuo 1997, Tanaka et al. 2010). Cross-breeding of rice started in 1921 in Japan, and thousands of cultivars have since been developed. Among them, Koshihikari is the preferred cultivar of Japanese consumers.

Koshihikari, a premium short-grain rice cultivar, was bred in 1956. It became the cultivar with the largest cultivation area in 1979, and since then, has continuously maintained its top position in Japan. In 2016, Koshihikari was planted on about 535,000 ha, which amounts to 36.2% of the total paddy rice area (Rice Stable Supply Support Organization Website, Ministry of Agriculture, Forestry and Fisheries Website-1). The main reason for this widespread preference for Koshihikari by Japanese consumers is its superior eating quality. Japanese people prefer a sticky and chewy texture, and dislike a dry and crisp texture. Strong stickiness is one of its most important features (Tanaka et al. 2006, Takeuchi et al. 2008, Wada et al. 2008). On the other hand, Koshihikari has two serious defects: low resistance to both lodging and rice blast. To extend cultivation of Koshihikari to wider areas, it was necessary to overcome those defects while retaining its high eating quality. Therefore, many agricultural researchers have studied Koshihikari deeply in terms of its ecology, morphological characteristics, physiological functions, cultivation techniques, eating quality, physicochemical properties, and genomics.

By the end of the twentieth century, Koshihikari was also cultivated outside of Japan. In California, USA, Koshihikari was cultivated on about 400 ha in 1991 (Iwata 1992). In New South Wales, Australia, Koshihikari has been cultivated since 1991, and the paddy yield was very high, at about 13.7 t/ha (Ohnishi et al. 1993). Koshihikari is also accepted by Chinese consumers; for example, Koshihikari rice produced in Japan was sold for about 50 times the price of ordinary Chinese rice at a Chinese supermarket in 2007 (Lee et al. 2010, Ministry of Agriculture, Forestry and Fisheries Website-2). It has also been produced by organic farming methods in Yu Tai Xian, Shandong Province, China, since 2016. Though people expect high eating quality from Koshihikari in these areas, environmental conditions such as high temperature and low humidity tend to prevent it from achieving the same quality as in Japan (Iwata 1992, Kawamura et al. 1996). Despite these drawbacks, breeding, research, and selection to improve the eating quality of Koshihikari (again, used as the standard of comparison) have been done energetically in China (Lee et al. 2010, Liang et al. 2013, Sun et al. 2006). The high seedling establishment of Koshihikari under both low temperature and deep-sowing conditions prompted the proposal to improve the direct-seeding suitability of Cambodian rice by using Koshihikari (Tong et al. 2007), in spite of its long culm and low resistance to lodging.

Although breeders have tried to mitigate or eliminate the defects of Koshihikari using it in breeding programs to produce rice with high eating quality, no cultivar is more widely cultivated than Koshihikari in Japan. This seems to be due to Koshihikari’s high brand recognition and the conservative eating preferences of Japanese consumers. However, in recent years, several cultivars with different eating quality have been developed that are being gradually accepted by consumers. In this review, we look back on the breeding history, dissemination, and characteristics of Koshihikari, then summarize genetic studies of its agronomic traits, as well as some improvements in Koshihikari. We conclude with a discussion of the future prospects for going beyond Koshihikari.

## Review

### Agricultural profile of Koshihikari

#### Breeding of Koshihikari

**Fig. 1:**
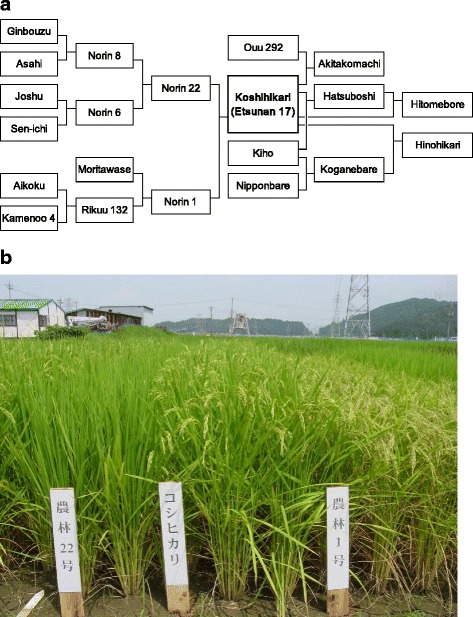
Pedigree of Koshihikari and its relatives. **a** Breeding history of Koshihikari (Etsunan 17). **b** Plant stature of Koshihikari (center) and its parents, Norin 22 (left) and Norin 1 (right)

Figure 1 shows the pedigree of Koshihikari and its relatives. Norin 22 and Norin 1 were crossed at the Niigata Prefectural Agricultural Experiment Station in 1944. Norin 1 was a mainstay cultivar in the Hokuriku region at that time owing to its early heading and high yield. However, rice breeders tried to reduce its susceptibility to blast disease by crossing it with Norin 22, which had the strongest resistance to rice blast disease at that time. The hybrid F_1_ seeds were cultivated in 1946, and 3000 F_2_ individuals were subsequently cultivated at the Nagaoka National Agricultural Experiment Station in Niigata Prefecture; 65 promising individuals were selected from this generation. In 1948, 20 lines of F_3_ material were transferred to the Fukui Agricultural Experiment Station. Unfortunately, a magnitude 7.1 earthquake hit the Fukui area on 28 June 1948; it completely destroyed the city, and most breeding materials at the station were buried under liquefied soil and lost. Fortunately, however, the F_3_ lines from Nagaoka survived, and five promising lines were selected. In 1949, F_4_ material from those lines was cultivated, and a superior line was selected.

**Fig. 2:**
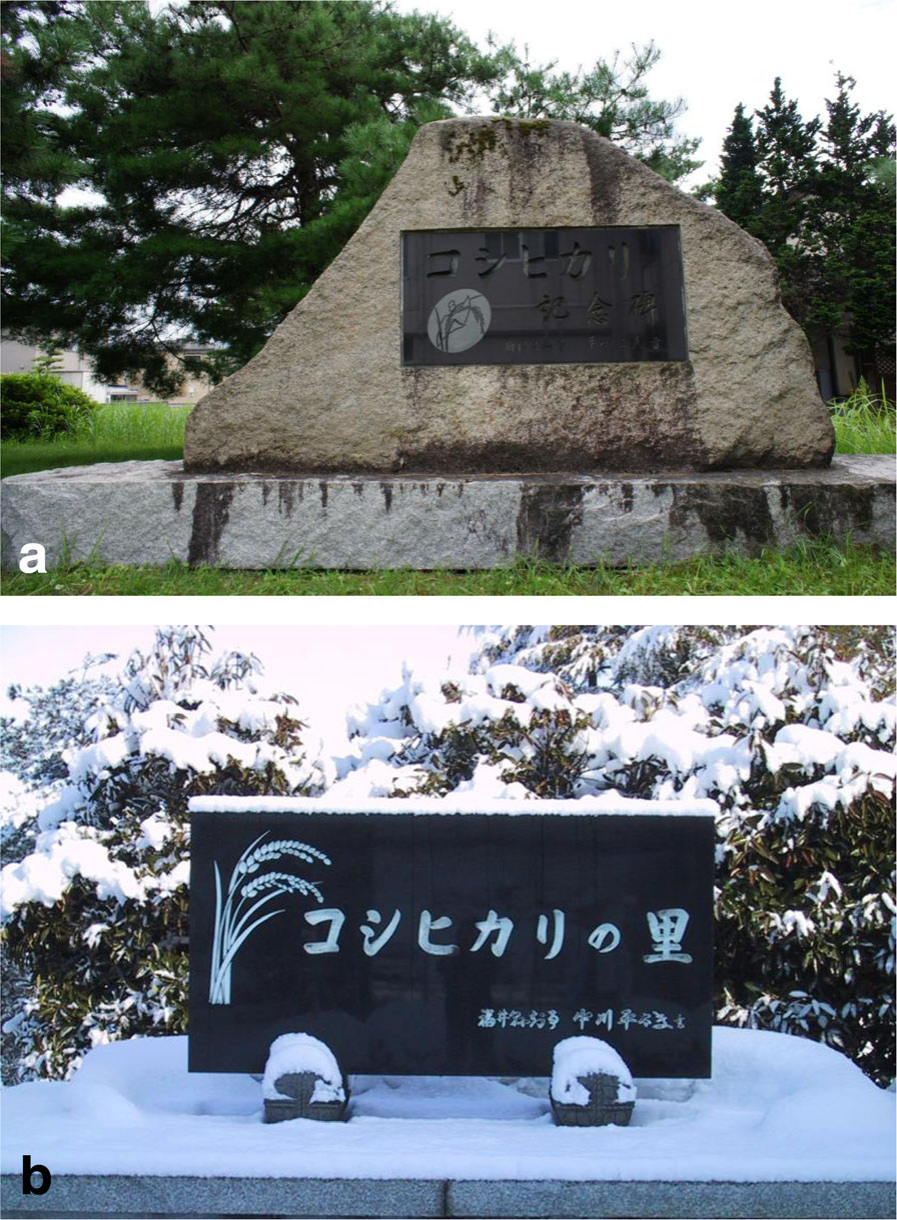
Stone monuments installed to commemorate the development of Koshihikari. Two prefectures, Niigata and Fukui, contributed to the development and dissemination of Koshihikari. **a** The monument in Niigata Prefecture (photo provided by the Niigata Agricultural Research Institute). **b** The monument in Fukui Prefecture (photo by Asako KOBAYASHI)

In 1953, an F_8_ line derived from the superior line was named Etsunan 17, and was released in 14 prefectures to examine its regional adaptability. In 1956, Etsunan 17 was adopted as a recommended cultivar by Niigata Prefecture and was renamed Koshihikari (Fig. 1). At the same time, Koshihikari was registered as an excellent cultivar by the Ministry of Agriculture, Forestry and Fisheries (Fukui Prefectural Government 1995). In addition to contributing to the breeding of Koshihikari, Niigata Prefecture played a leading role during its dissemination and in the establishment of cultivation methods, both of which are described in the following sections. Stone monuments have been created to commemorate the development of Koshihikari in Niigata and Fukui prefectures (Fig. 2).

#### Dissemination history

**Fig. 3:**
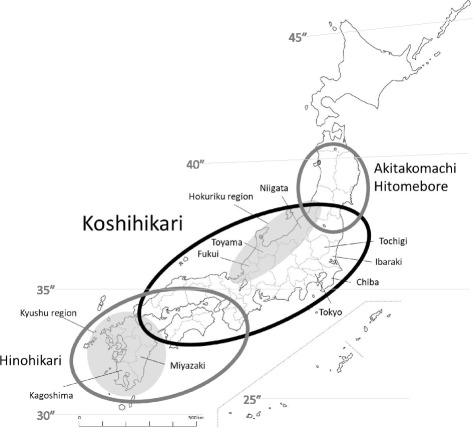
Main areas where Koshihikari and its progeny are cultivated in Japan

**Fig. 4:**
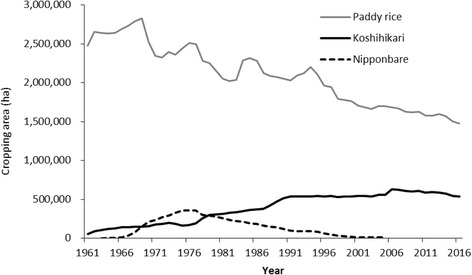
Cultivation areas of total paddy rice, Koshihikari, and Nipponbare in Japan. Data was obtained from the Rice Stable Supply Support Organization Website and National Agriculture and Food Research Organization Website

Around the same time or just after adoption by Niigata Prefecture in 1956, three prefectures close to Tokyo—Chiba, Tochigi, and Ibaraki (Fig. 3)—adopted Koshihikari as one of their recommended cultivars in 1956, 1957, and 1959, respectively. They were aiming to sell it to Tokyo, the largest consumption area in Japan. Kagoshima Prefecture adopted it in 1960, followed by Miyazaki Prefecture in 1961. Since these two prefectures are in a warmer region of Japan (Fig. 3), they have an advantage at producing competitive rice because of earlier harvest each year. The abundant rice harvests from 1966 to 1969 brought an era of surplus rice. In 1969, the government held 553 Mt of old rice stock in its reserves. As a result, the nature of the demand for rice changed dramatically, from an emphasis on quantity to an emphasis on quality. Koshihikari rice met this demand. In the 1970s, prefectures in western Japan and in the Hokuriku region adopted Koshihikari as their recommended cultivar to meet this demand. Koshihikari’s share of the total cultivated area became the largest of any cultivar in Japan in 1979, reaching 297,000 ha (13.2% of the total), and it has continuously maintained the top position since then (Fig. 4). The maximum area of Koshihikari was recorded in 2006, at about 640,000 ha. Japanese rice consumption has decreased more or less continuously since the 1970s, at a rate of about 80 kt per year (Rice Stable Supply Support Organization Website), and Koshihikari began to follow this trend in 2006.

In 1956, the average paddy yield of Koshihikari ranged from 4940 to 5440 kg/ha according to an unpublished breeding report by the Fukui Agricultural Experiment Station. During the cultivar’s dissemination throughout Japan, many cultivation techniques have been developed to prevent lodging, increase yield, and retain the high eating quality. These include transplanting of younger seedlings to increase the duration of the growing season (Horiguchi et al. 1991); improving water management by allowing soil drying during growth to suppress excessive culm elongation; diagnosing growth problems using the culm length, leaf color, and culm number (Sasaki 1985, Ichimaru and Kanayama 1989); splitting the application of fertilizer during the reproductive stage (Matsuzaki et al. 1982, Ikeda and Kasai 1986, Sasaki 1985); and using growth regulators (Hashizume and Yamagishi 1969). As a result, the paddy yield of Koshihikari increased to 6630 kg/ha by 1985 in the Hokuriku region. Recently, delayed transplanting time (Yamaguchi et al. 2004, Morita et al. 2011) and the use of deep irrigation (Chiba et al. 2011) have been adopted to avoid heat-induced declines in the quality of rice kernels during the ripening period. Direct seeding of Koshihikari has been tried, but it has large disadvantages because it produces long culms and a higher lodging rate (Sakai et al. 2000, Sato and Sakai 2004).

#### Agronomic characteristics of Koshihikari

Japan covers a wide range of latitude, from 46°N to 20°N, so environmental characteristics such as day length and temperature vary widely among regions. Therefore, rice cultivars are usually chosen to fit the environment of each area. However, Koshihikari can be cultivated at latitudes ranging from 40°N to 31°N (Fig. 3). This high adaptability of Koshihikari originates from its heading properties. In addition, the basic vegetative growth duration of Koshihikari was 35.2 days when it was cultivated under short-day conditions after sprouting at 28 °C (Mimoto et al. 1989). This duration could be divided into the juvenile phase, when it is insensitive to the short-day conditions (13.1 days), and the reproductive phase until heading, when it is sensitive to photoperiod (22.1 days). Its temperature sensitivity is high (Hosoi 1979), and its photoperiod sensitivity is relatively weak compared with other Japanese rice cultivars (Mimoto et al. 1989, Hori et al. 2013).

It is noteworthy that the advantages of Koshihikari have gradually become clear during its dissemination throughout Japan. Its strong cold tolerance during the booting stage was identified in paddy fields irrigated with cold water in northern regions (Ozeki et al. 1995), and Hosoi (1989) found that this was relatively strong on the basis of the critical low temperature at which a cultivar is able to produce at least 80% of perfectly mature grains, although Koshihikari was selected without a prior evaluation for cold tolerance. This strong cold tolerance during the booting stage was inherited by Hitomebore and Akitakomachi (Fig. 1, Fig. 3); these cultivars have frequently resisted damage from cold weather in northern regions of Japan.

Koshihikari’s strong tolerance to pre-harvest sprouting was revealed in the southern Kyushu region, where harvesting sometimes occurs during the rainy season (early July). This trait has also been very helpful in Hokuriku, where rice is also harvested during the rainy season (September) (Ishizaka et al. 1989).

High eating quality is one of the major reasons why Koshihikari is preferred by Japanese consumers. The eating quality of Koshihikari is superior to that of Nipponbare, a standard rice, in appearance, scent, flavor, stickiness, and overall evaluation, and cooked Koshihikari rice is much softer than Nipponbare (Takeuchi et al. 2008). Its eating quality was evaluated as the highest among 112 cultivars in Japan (Yamamoto and Ogawa 1992). The amylose content of Koshihikari is relatively low (Koike et al. 1993); in Fukui Prefecture from 1994 to 2007, it averaged 17.2%, versus average of 19.4% for Nipponbare. Cooked rice grains of Koshihikari show strong stickiness (Tanaka et al. 2006, Kobayashi et al. 2008, Takeuchi et al. 2008, Wada et al. 2008). This feature has been inherited by other Japanese rice cultivars such as Hitomebore and Akitakomachi that are progeny of Koshihikari (Fig. 1).

Nevertheless, Koshihikari has disadvantages. Its lodging resistance was rated as ‘very weak’ (Takaya and Miyasaka 1981, Terashima et al. 1992) owing to its long culm and its low breaking strength (Ookawa and Ishihara 1992). Its blast resistance in the field was rated as ‘weak’ for leaf blast and ‘slightly weak’ for panicle blast (Ishizaka et al. 1989); although Koshihikari has some resistance genes, including *Pish* and *Pik-s*, blast races virulent against *Pish* and *Pik-s* are distributed widely in Japan (Kawasaki-Tanaka and Fukuta 2014, Kawasaki-Tanaka et al. 2016). It is susceptible to the leaf stripe virus and shows slightly strong resistance to bacterial leaf blight (Ishizaka et al. 1989, Ogawa et al. 1998). Koshihikari has medium incidence of bacterial brown stripe disease (Kadota and Ohuchi 1989). In addition, with global warming, average dairy minimum temperature from June to August has increased by about 1.1 °C from 1966 to 2001 (Kawatsu et al. 2007), and temperature during ripening period has also increased by 1.1 °C above that in past years (Sugiura et al. 2013). Therefore, a heat-induced decline in the quality of Koshihikari has been observed in many prefectures (Sugiura et al. 2013, Inoue 2012).

#### Progeny of Koshihikari

Table 1 shows the share of the cultivated area by Japan’s top 10 paddy rice cultivars in 2016. The three top-ranked cultivars after Koshihikari (Hitomebore, Hinohikari, and Akitakomachi) are all direct descendants of Koshihikari (Fig. 1). The remaining four cultivars (Nanatsuboshi, Haenuki, Kinuhikari, and Massigura) are also descendants of Koshihikari. They can be classified as Koshihikari-like in terms of their eating quality. The top 10 cultivars account for 75.6% of the total rice production area in Japan. At present, 104 cultivars have been bred using Koshihikari as a direct mating parent by many of the breeding divisions of prefectural and national research institutes (National Agriculture and Food Research Organization Website).

**Table 1:** Rice cultivation in 2016 in Japan (source: Rice Stable Supply Support Organization website)

No.	Variety	Share (%)	Cross	Year developed
1	Koshihikari	36.2	–	1956
2	Hitomebore	9.6	Koshihikari/Hatsuboshi	1991
3	Hinohikari	9.1	Kobanebare/Koshihikari	1989
4	Akitakomachi	7.0	Koshihikari/Ouu 292	1984
5	Nanatsuboshi	3.5	Hitomebore/Kukei 90242A//Kuiku 150	2001
6	Haenuki	2.8	Shonai 29/Akitakomachi	1991
7	Kinuhikari	2.5	Shu 2800/Hokuriku 100//Hokuriku 96	1988
8	Massigura	1.8	Ouu 341/Yamagata 40	2005
9	Asahi no yume	1.6	Aichi 70//Aichi 56/Aichi 65	1996
10	Yumepirika	1.5	Satsukei 96118/Jouiku 427	2008
Total		75.6		–

Hokuriku 100: gamma-ray mutant of Koshihikari

Shu 2800: a descendant of IR8 and Koshihikari

Ouu 341: a grandchild of Koshihikari

Koshihikari is a valuable genetic resource for good eating quality, but its long culm and susceptibility to rice blast disease have often caused difficulty for its use as a parent. To compensate for these disadvantages, mutation and backcross breeding of Koshihikari have been used frequently. At present, 29 cultivars have been developed by mutation of Koshihikari (National Agriculture and Food Research Organization Website). Hokuriku 100, a semi-dwarf mutant of Koshihikari, was bred by means of gamma-ray mutation, and has a 30% shorter culm length than Koshihikari (Tanisaka et al. 1990). Jikei 58 was selected from protoplast-derived plants of Koshihikari, and has a 20% shorter culm length (Noda et al. 1997). Mutations for the physicochemical components of kernel quality of Koshihikari have also been selected. A dull-white mutant, Milky Queen, was selected from the progeny after treatment of Koshihikari with N-methyl-N-nitrosourea; it has a lower amylose content (9% to 12%) in the endosperm than Koshihikari (17.5%) (Ise et al. 2001). Yumegokochi was selected from the progeny of protoplast-derived plants of Koshihikari and also has a 1.5% lower amylose content (Sukekiyo et al. 1995). Using gamma-ray mutation, rice cultivars have been bred that lack alpha-globulin protein (Iida et al. 1998, Nishimura et al. 2005), and a strong culm (Samoto and Kanai 1975). A novel cultivar with low cadmium levels in its endosperm, Koshihikari Kan No. 1, was selected from mutations induced by ion-beam irradiation (Ishikawa et al. 2012, Abe et al. 2017).

At present, 55 isogenic and near-isogenic lines (NILs) of Koshihikari have been developed (National Agriculture and Food Research Organization Website). Notably, blast-resistant NILs possessing one or more *R*-genes in the Koshihikari genetic background were developed: Kojima et al. (2003) developed six NILs in Toyama Prefecture; Ishizaki et al. (2005) developed 12 NILs in Niigata Prefecture. Seeds of these NILs are now mixed in a ratio that is determined from disease forecasts by field pathologists, and are provided to the farmers as a BL series (i.e., blast-resistant lines) every year. Accessions in the BL series are designated as lines that belong to the Koshihikari brand. About 88,000 ha of cultivation of these accessions throughout Niigata Prefecture has decreased the occurrence of rice blast, and this has let farmers reduce pesticide use by 25% (Ishizaki et al. 2005, Niigata Prefecture Website). To increase lodging resistance, backcross breeding to incorporate one or more semi-dwarfing genes has been carried out at several universities and local rice research institutes (Nonaka et al. 1991, Murai and Endo 2006, Tomita 2009). However, these cultivars have not replaced the original Koshihikari in most places, because some skillful farmers have resolved the lodging problem and prioritize current Koshihikari over “semi-dwarf” Koshihikari.

### Genomics of Koshihikari

#### Genome resequencing and haplotype inheritance

The rice genome sequence was decoded in 2005 (IRGSP 2005). After this breakthrough, many of the agriculturally important genes were identified by advanced research institutes around the world. In parallel, researchers have aimed to understand the breeding history and characteristics of Koshihikari by using DNA markers.

Yamamoto et al. (2010) determined the whole-genome sequence of Koshihikari by using next-generation sequencing. Though the average short read length of next-generation sequencing was 30 to 35 base pairs, these authors aligned the reads and completed a pseudomolecule of Koshihikari by using the Nipponbare genome sequence as a reference. They estimated 67,051 single-nucleotide polymorphisms (SNPs) between these two cultivars. A GoldenGate typing array consisting of 1917 SNPs extracted from this total revealed genome-wide polymorphisms in 151 Japanese cultivars. By comparing the polymorphism information with pedigree records of rice breeding in Japan, Yamamoto et al. (2010) identified the ancestral origin of the pedigree haplotypes in 60.9% of the Koshihikari genome. Furthermore, they identified 28.6% of Koshihikari’s ancestral landraces. The proportion of the Koshihikari genome inherited by three of its famous progeny―Hitomebore, Akitakomachi, and Hinohikari―was 80.8, 80.0, and 61.3%, respectively. In addition, 18 pedigree haplotypes were inherited from landraces and shared among Koshihikari and these three progeny, which together account for 61.9% of the total cultivated area in Japan (Table 1).

As we noted in the previous section, rice breeders tend to use Koshihikari and select phenotypes, especially for good eating quality, that resemble it. This may explain the wide dissemination of Koshihikari’s genome, which suggests the low frequency of DNA polymorphisms among recent Japanese cultivars. However, information on the large number of SNPs obtained from the whole-genome sequence overcame this inefficiency of DNA marker development, and has accelerated the detection of agronomically important quantitative trait loci (QTLs).

#### QTL analysis in experimental populations

Detection of QTLs is a primary step in efforts to understand the genetic basis for agronomic traits, because many agronomic traits are generally controlled by multiple QTLs. To identify the QTLs involved in the control of agronomically important traits in Koshihikari and Nipponbare, researchers have developed two types of segregating populations—backcross inbred lines (BILs) and chromosome segment substitution lines (CSSLs)—from reciprocal crosses between the two cultivars (Matsubara et al. 2008, Hori et al. 2010).

**Fig. 5:**
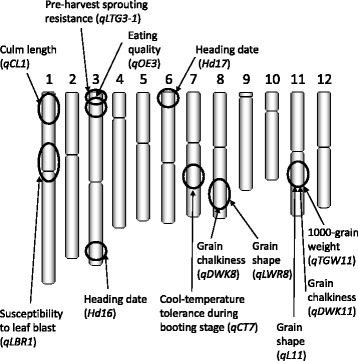
Positions of quantitative trait loci (QTLs) associated with agronomic traits in Koshihikari. See the text for the sources of these discoveries

By using the BILs and CSSLs, major-effect QTLs were detected for several agronomic traits, including heading date, eating quality, strong tolerance to pre-harvest sprouting, tolerance to cool temperatures during the booting stage, grain shape, grain chalkiness, culm length, 1000-grain weight, and leaf blast resistance in Koshihikari (Fig. 5) (Matsubara et al. 2008, Takeuchi et al. 2008, Hori et al. 2009, Hori et al. 2010, Hori et al. 2012, Tanabata et al. 2012). The genetic effects of each QTL have been confirmed in advanced backcross populations, and two novel heading date genes (*Hd16* and *Hd17*) have been cloned by means of map-based strategies (Matsubara et al. 2012, Hori et al. 2013, Hori et al. 2017). *Hd16* is involved in the control of photoperiod sensitivity in rice (Hori et al. 2013). During the process of rice introduction at higher latitudes, breeders have selected lines with weaker sensitivity to photoperiod to produce an early heading date so as to ensure maturity during the period of optimal climatic conditions. Because the Koshihikari *Hd16* allele produced weak photoperiod sensitivity and an early heading date, acquisition of this allele may have permitted extension of the cultivation area for Koshihikari into more northern and southern cultivation areas in Japan. *Hd17* is also involved in the control of photoperiod sensitivity (Matsubara et al. 2012). *Hd17* may have been used to modify heading date slightly in rice cultivars, because its genetic effect is small.

A QTL for good eating quality in Koshihikari was detected on the short arm of chromosome 3 (Takeuchi et al. 2008, Hori and Yano 2013). Other genetic studies have found major-effect QTLs for eating quality traits by using segregating populations derived from crosses with Koshihikari (Takeuchi et al. 2007, Kobayashi et al. 2008, Wada et al. 2008). Fine-mapping of a seed dormancy QTL revealed that a nonfunctional *qLTG3–1* allele, was associated with strong tolerance to pre-harvest sprouting in Koshihikari (Hori et al. 2010). A novel QTL on the short arm of chromosome 1 (*qCL1*) was associated with long culm length and weak lodging resistance in Koshihikari (Hori et al. 2009, Hori et al. 2012). A QTL for strong cold tolerance at the booting stage (*qCT7*) was detected on the long arm of chromosome 7 (Takeuchi et al. 2001, Hori et al. 2017). Fukuoka et al. (2010) and Hori et al. (2017) detected and validated the QTL for leaf blast resistance (*qLBR1*) on the short arm of chromosome 1. This QTL allele in Koshihikari conferred susceptibility to leaf blast infection. These QTLs contributed strongly to agronomic characteristics in Koshihikari.

#### Marker-assisted breeding for “pinpoint” improvement of Koshihikari

To increase the agronomic value of Koshihikari, many researchers have tried to introduce new genes from diverse genetic resources into it. Several series of CSSLs using Koshihikari as the recurrent parent have contributed greatly to this research (Ebitani et al. 2005, Takai et al. 2007, Takai et al. 2014, Abe et al. 2013, Murata et al. 2014, Nagata et al. 2015). Because a large amount of information about the environmental responses of Koshihikari has accumulated, a small but stable effect on a target trait can be accurately evaluated. This means that breeders who use Koshihikari can take advantage of high-resolution marker-assisted breeding (“pinpoint” improvement) by using NILs. Some CSSLs have been derived from wild relatives such as *Oryza rufipogon, O. glumaepatula, and O. nivara* (Hirabayashi et al. 2010, Furuta et al. 2014, Furuta et al. 2016). Ashikari et al. (2005) detected QTLs for grain number and culm length from a high-yielding *indica* cultivar in the Koshihikari genetic background, and introduced them into Koshihikari to create a new line with both high yield and higher lodging resistance.

Since this successful QTL pyramiding, many trials of pinpoint improvement of Koshihikari have been reported (category A in Additional file 1: Table S1). Though the target QTLs were not cloned, their phenotypic effects in the Koshihikari genetic background have been evaluated and incorporated in actual breeding programs (categories B and C in Additional file 1: Table S1). In addition to successful trials of a Koshihikari BL series (Ishizaki et al. 2005) described earlier in this paper, improvement of panicle blast resistance (Sugiura et al. 2004) and development of a semi-dwarf phenotype (Wang et al. 2005) were also achieved by using DNA markers. However, pinpoint improvement of other agronomic traits, especially yield-related traits, is still in progress or under evaluation. This work has not encountered significant technical problems, but the improved cultivars have not always proven to be acceptable to Japanese farmers or consumers. Some problems, such as tight linkage, pleiotropy or introgression out of the target region, need to be overcome. The incredibly strong power of the Koshihikari brand has been a major barrier to dissemination of these new cultivars, even though they are NILs of Koshihikari.

### Future perspectives for Koshihikari research

Table 1 shows the continuing preference of Japanese consumers for cooked Koshihikari rice: the top five cultivars were developed more than 15 years ago, and Koshihikari-like sticky rice has been loved since the 1950s. However, Japanese rice consumption has been decreasing since the 1970s, by 80 kt per year (Rice Stable Supply Support Organization Website). This has resulted from Japan’s decreasing population, which has resulted from a declining birthrate and the aging population, and has been exacerbated by a transition to increased consumption of wheat products such as bread. Furthermore, there has been increasingly wide diversification of the eating quality of cooked rice in Japan. For example, younger Japanese tend to prefer harder cooked rice rather than sticky rice like Koshihikari according to recent market research (Fukui Prefecture, unpublished data). To meet these demands, Japanese rice breeders have tried to develop new cultivars that can increase rice consumption and respond to the diversification of eating quality preferences.

One solution has been the development of rice with different eating qualities from Koshihikari. For example, Ichihomare (Kobayashi et al. 2017), Shinnosuke (Kasaneyama et al. 2017), and Tsuyahime (Asanome et al. 2010) have a somewhat hard surface in the cooked rice and are being accepted as a new type of rice by consumers. In contrast, Yumepirika (Yoshida et al. 2013) and Pikamaru (Sakai et al. 2016) have a low amylose content (16.1% and about 10%, respectively) and their cooked rice is stickier than Koshihikari. In addition, the appearance of cooked rice is one of the most important characteristics for judgments of eating quality (Oosato et al. 1998, Shigemune et al. 2007). Objective measurements of the whiteness of cooked rice have been obtained using a spectrophotometer (Goto et al. 2014) or by means of image analysis (Kogi et al. 2014). A few cultivars are whiter and glossier than Koshihikari (Machida et al. 2017).

About 30% of rice consumption is accounted for by take-home rice from supermarkets and eating in restaurants (Rice Stable Supply Support Organization Website). Unique properties are needed for rice that is processed by large-scale rice cooking systems in factories; these include a somewhat hard surface for the cooked rice, a volume increase after cooking, slower degradation during refrigerated transport, and suitability for blending with other rice cultivars; unfortunately, these properties are not provided by Koshihikari rice. Recently, several Japanese rice breeding projects have attempted to develop cultivars with such properties to support industrial uses. These projects have been started by the National Agriculture and Food Research Organization and prefectural governments to meet current and future demands.

Another solution is “healthy” rice. Recently, health consciousness has been increasing in Japan. Researchers have tried to take advantage of Koshihikari’s good eating quality while adding functionality. Idesawa et al. (2017) selected a line that has 2 to 3.1 times the content of dietary fiber in the endosperm of Koshihikari using progeny derived from gamma-irradiation. Black (Maeda et al. 2014) and red (Yamaguchi et al. 2015) rice contain anthocyanins and tannins (both healthful nutraceuticals), respectively, and these cultivars were developed using Koshihikari as a recurrent parent. A mutant line of Koshihikari that was deficient in a 26-kDa globulin was used to develop cultivars, with a low content of easy-to-digest protein (Iida et al. 1998, Nishimura et al. 2005).

Breeders have continued to develop new rice varieties whose properties surpass those of Koshihikari. Scientists have continued to elucidate the secrets of Koshihikari’s genome. Farmers have continued to provide delicious rice, including both Koshihikari and cultivars with different characteristics that meet the demands of consumers. Such sincere and large-scale efforts will continue, leading to sustainable rice production in Japan’s paddy fields.

## Conclusions

Despite weak resistance to blast diseases and lodging susceptibility, Japanese farmers have learned to successfully cultivate Koshihikari by applying their practical skills, leading to widespread adoption of Koshihikari. Japanese manufacturers have also been involved in rice cultivation, processing, and cooking to adapt their products to use Koshihikari. In addition, the spread of Japanese food restaurants outside of Japan has accelerated demand for rice with high eating quality. These trends have increased the value of Koshihikari and Koshihikari-like rice. For these reasons, rice-using industries, scientists, and consumers can’t discuss Japanese rice without thinking of Koshihikari.

However, the dominance of Koshihikari or its genome caused by excessive reliance on this cultivar has not always been beneficial for rice in Japan. As the national demographic structure and Japanese lifestyles and preferences change, the nature of the demand for rice is also changing. It is also necessary to prepare a broader range of genetic materials to protect against both expected and unexpected environmental changes, such as global warming, soil degradation, and typhoons. The rapid decrease in the number of farmers is a serious social concern in Japan, and suggests a need for rice that can be cultivated more easily by the remaining farmers. For all these reasons, development of rice cultivars with high and sustainable yield, combined with low cultivation costs, is an urgent breeding objective. In this context, improving our understanding of the genetic basis of Koshihikari’s agronomic characteristics, followed by comprehensive utilization of this knowledge for genetic improvement, will be more important than ever before.

## Additional file

Additional file 1: **Table S1.** Genetic studies using Koshihikari as recurrent parent with the goal of improving the agronomic characteristics of Koshihikari. Category A: isolated as single-gene locus, and phenotype proven in the Koshihikari genetic background. Category B: QTL delimited but unidentified in the Koshihikari genetic background. Category C: comprehensive genome-wide analysis by Koshihikari genetic background. (DOCX 23 kb)

## Abbreviations

BIL: Backcross inbred line
BL: Blast-resistant line
CSSL: Chromosome segment substitution line
NIL: Near-isogenic line
QTL: Quantitative trait locus
SNP: Single-nucleotide polymorphism
