# Supplementary material for: Koshihikari: a premium short-grain rice cultivar – its expansion and breeding in Japan
Source: Rice (N Y). 2018 Apr 9;11:15. doi: 10.1186/s12284-018-0207-4 (PMC5890008; doi:10.1186/s12284-018-0207-4)
Supplement: Supplementary file 1 — Table S1. Genetic studies using Koshihikari as recurrent parent with the goal of improving the agronomic characteristics of Koshihikari. Category A: isolated as single-gene locus, and phenotype proven in the Koshihikari genetic background. Category B: QTL delimited but unidentified in the Koshihikari genetic background. Category C: comprehensive genome-wide analysis by Koshihikari genetic background. (DOCX 23 kb) [file 12284_2018_207_MOESM1_ESM.docx]

**Supplemental Table S1**. Genetic studies using Koshihikari as recurrent parent with the goal of improving the agronomic characteristics of Koshihikari. Category A: isolated as single-gene locus, and phenotype proven in the Koshihikari genetic background. Category B: QTL delimited but unidentified in the Koshihikari genetic background. Category C: comprehensive genome-wide analysis by Koshihikari genetic background.

| Category | Trait | Donor | Locus name | Chromosome | Note | Reference |
| --- | --- | --- | --- | --- | --- | --- |
| A | blast resistance, rice stripe virus resistance | Modan | *Pb1, Stvb-i* | 11 | Os11g0505300, Os11g0444700 | Sugiura et al. (2004) |
|  | grain number | Habataki | *Gn1a(OsCKX2)* | 1 | Os01g0197700 | Ashikari et al. (2005) |
|  | regeneration ability | Kasalath | *PSR1* | 1 | Os01g0357100 | Nishimura A et al. (2005) |
|  | semi-dwarf | IR24 | *sd1(GA20ox2)* | 1 | Os01g0883800 | Wang et al. (2005) |
|  | heading date | Kasalath | *Hd1, Hd4, Hd5, Hd6* | 6,7,8,3 | Os06g0275000, Os07g0261200, Os08g0174500, Os03g0762000 | Takeuchi et al. (2006) |
|  | lodging resistance | Habataki | *SCM2(APO1)* | 6 | Os06g0665400 | Ookawa et al. (2010) |
|  | sink and source capacity | Kasalath | *tgw6* | 6 | Os06g0623700 | Ishimaru et al. (2013) |
|  | leaf photosynthesis | Takanari | *GPS(NAL1)* | 4 | Os04g0615000 | Takai et al. (2013) |
|  | lodging resistance | Chugoku117 | *SCM3(OsTB1)* | 2 | Os03g0706500 | Yano et al. (2014) |
|  | leaf photosynthesis | Habataki | *CAR8(DTH8/Ghd8/LHD1)* | 8 | Os08g0174500 | Adachi et al. (2017) |
| B | rice stripe virus resistance | Kanto72 | *STV2, STV11* | 11 |  | Maeda et al. (2006) |
|  | stigma exsertion | IR24 | *qES3* | 3 |  | Miyata et al. (2007) |
|  | lodging resistance | Kasalath | *lrt5* | 5 |  | Ishimaru et al. (2008), Hirotsu et al. (2010) |
|  | nitrogen use efficiency | Kasalath | *qRL6.1* | 6 |  | Obara et al. (2010) |
|  | seed germination under low temperature | Awa-akamai | *qESS11* | 11 |  | Yamaguchi et al. (2010) |
|  | low cadmium content | Jarjan | *qCdp7* | 7 | Paper of BIL development | Abe et al. (2011) |
|  | low cadmium content | LAC23 | *qlGCd3* | 3 | Paper of CSSL development | Abe et al. (2013) |
|  | sheath blight resistance | Jarjan | *qSBR-9* | 9 |  | Taguchi-Shiobara et al. (2013) |
|  | bacterial blight resistance | NonaBokra | *qRBS1* | 10 |  | Mizobuchi et al. (2013) |
|  | high temperature tolerance | Habataki | *Apq1* | 7 | Paper of CSSL development | Murata et al. (2014) |
|  | colored rice | Hong Xie Nuo | *Kala1, Kala3, Kala4* | 1,3,4 |  | Maeda et al. (2014) |
|  | blast resistance | IRBL9-W | *Pi9* | 6 |  | Tsunematsu et al. (2015) |
|  | brown spot resistance | Tadukan | *qBSfR11* | 11 |  | Sato et al. (2015) |
|  | exudation rate at ripening | Akenohoshi | *qEXR1* | 2 | Paper of RIL development | Yamamoto et al. (2016) |
|  | leaf color degradation at ripening | Akenohoshi | *qCHR1* | 3 |  | Yamamoto et al. (2017) |
| C | heading date, plant height | Kasalath |  |  | Paper of CSSL development | Ebitani et al. (2005) |
|  | heading date | NonaBokra |  |  | Paper of CSSL development | Takai et al. (2007) |
|  | yield and its components | Kasalath |  |  |  | Madoka et al. (2008) |
|  | elongation rate | Kasalath |  |  |  | Hirotsu et al. (2008) |
|  | blast resistance | *Oryza rufipogon* |  |  | Paper of CSSL development | Hirabayashi et al. (2010) |
|  | yield and its components | *Oryza glaberrima* |  |  | Paper of CSSL development | Shim et al. (2010) |
|  | yield and its components | Kasalath, NonaBokra |  |  |  | Ujiie et al. (2012) |
|  | leaf area | Kasalath, NonaBokra |  |  |  | Ujiie et al. (2013) |
|  | yield and its components | Takanari |  |  | Paper of CSSL development | Takai et al. (2014) |
|  | yield and its components | *Oryza rufipogon* |  |  | Paper of CSSL development | Furuta et al. (2014) |
|  | grain shape | IR64 |  |  | Paper of CSSL development | Nagata et al. (2015) |
|  | yield and its components | IR64 |  |  |  | Ujiie et al. (2016) |
|  | lodging resistance | Takanari |  |  |  | Ookawa et al. (2016) |
|  | yield and its components | *Oryza nivara* |  |  | Paper of CSSL development | Furuta et al. (2016) |

**References**

Abe T, Taguchi-Shiobara F, Kojima Y, Ebitani T, Kuramata M, Yamamoto T, Yano M, Ishikawa S (2011) Detection of a QTL for accumulating Cd in rice that enables efficient Cd phytoextraction from soil. Breed Sci 61:43-51

Abe T, Nonoue Y, Ono N, Omoteno M, Kuramata M, Fukuoka S, Yamamoto T, Yano M, Ishikawa S (2013) Detection of QTLs to reduce cadmium content in rice grains using LAC23/Koshihikari chromosome segment substitution lines. Breed Sci 63:284-291

Adachi S, Yoshikawa K, Yamanouchi U, Tanabata T, Sun J, Ookawa T, Yamamoto T, Sage RF, Hirasawa T, Yonemaru J (2017) Fine mapping of Carbon Assimilation Rate 8, a quantitative trait locus for flag leaf nitrogen content, stomatal conductance and photosynthesis in rice. Front Plant Sci 8:60

Ashikari M, Sakakibara H, Lin S, Yamamoto T, Takashi T, Nishimura A, Angeles ER, Qian Q, Kitano H, Matsuoka M (2005) Cytokinin oxidase regulates rice grain production. Science 309:741-745

Ebitani T, Takeuchi Y, Nonoue Y, Yamamoto T, Takeuchi K, Yano M (2005) Construction and evaluation of chromosome segment substitution lines carrying overlapping chromosome segments of indica rice cultivar ‘Kasalath’ in a genetic background of Japonica elite cultivar ‘Koshihikari’. Breed Sci 55:65-73

Furuta T, Uehara K, Angeles-Shim RB, Shim J, Ashikari M, Takashi T (2014) Development and evaluation of chromosome segment substitution lines (CSSLs) carrying chromosome segments derived from Oryza rufipogon in the genetic background of Oryza sativa L. Breed Sci 63:468–475

Furuta T, Uehara K, Shim R, Shim J, Nagai K, Ashikari M, Takashi T (2016) Development of chromosome segment substitution lines harboring Oryza nivara genomic segments in Koshihikari and evaluation of yield-related traits. Breed Sci 66:845-850

Hirabayashi H, Sato H, Nonoue Y, Kuno-Takemoto Y, Takeuchi Y, Kato H, Nemoto H, Ogawa T, Yano M, Imbe T et al. (2010) Development of introgression lines derived from Oryza rufipogon and O. glumaepatula in the genetic background of japonica cultivated rice (O. sativa L.) and evaluation of resistance to rice blast. Breed Sci 60:604–612

Hirotsu N, Kashiwagi T, Madoka Y, Ishimaru K (2008) Time-related identification of chromosome regions affecting plant elongation in rice (Oryza sativa L.). Plant Physiol Biochem 46:517-523

Hirotsu N, Murakami N, Kashiwagi T, Ujiie K, Ishimaru K (2010) Protocol: a simple gel-free method for SNP genotyping using allele-specific primers in rice and other plant species. Plant Methods 6:12

Ishimaru K, Togawa E, Ookawa T, Kashiwagi T, Madoka Y, Hirotsu N (2008) New target for rice lodging resistance and its effect in a typhoon. Planta 227:601–609

Ishimaru K, Hirotsu N, Madoka Y, Murakami N, Hara N, Onodera H, Kashiwagi T, Ujiie K, Shimizu B, Onishi A et al. (2013) Loss of function of the IAA-glucose hydrolase gene TGW6 enhances rice grain weight and increases yield. Nat Genet 45:707–711

Madoka Y, Kashiwagi T, Hirotsu N, Ishimaru K (2008) Indian rice “Kasalath” contains genes that improve traits of Japanese premium rice “Koshihikari”. Theor Appl Genet 116:603-612

Maeda H, Matsushita K, Iida S, Sunohara Y (2006) Characterization of two QTLs controlling resistance to rice stripe virus detected in a Japanese upland rice line, Kanto 72. Breed Sci 56:359-364

Maeda H, Yamaguchi T, Omoteno M, Takarada T, Fujita K, Murata K, Iyama Y, Kojima Y, Morikawa M, Ozaki H et al. (2014) Genetic dissection of black grain rice by the development of a near isogenic line. Breed Sci 64:134-141

Miyata M, Yamamoto T, Komori T, Nitta N (2007) Marker-assisted selection and evaluation of the QTL for stigma exsertion under japonica rice genetic background. Theor Appl Genet 114:539-548

Mizobuchi R, Sato H, Fukuoka S, Tsushima S, Imbe T, Yano M (2013) Identification of qRBS1, a QTL involved in resistance to bacterial seedling rot in rice. Theor Appl Genet 126:2417–2425

Murata K, Iyama Y, Yamaguchi T, Ozaki H, Kidani Y, Ebitani T (2014) Identification of a novel gene (Apq1) from the indica rice cultivar “Habataki” that improves the quality of grains produced under high temperature stress. Breed Sci 64:273–281

Nagata K, Ando T, Nonoue Y, Mizubayashi T, Kitazawa N, Shomura A, Matsubara K, Ono N, Mizobuchi R, Shibaya T, Ogiso-Tanaka E, Hori K, Yano M, Fukuoka S (2015) Advanced backcross QTL analysis reveals complicated genetic control of rice grain shape in a japonica × indica cross. Breed Sci 65:308–318

Nishimura A, Ashikari M, Lin S, Takashi T, Angeles ER, Yamamoto T, Matsuoka M (2005) Isolation of a rice regeneration quantitative trait loci gene and its application to transformation systems. Proc Natl Acad Sci USA 102:11940-11944

Obara M, Tamura W, Ebitani T, Yano M, Sato T, Yamaya T (2010) Fine-mapping of qRL6.1, a major QTL for root length of rice seedlings grown under a wide range of NH4+ concentrations in hydroponic conditions. Theor Appl Genet 121:535–547

Ookawa T, Hobo T, Yano M, Murata K, Ando T, Miura H, Asano K, Ochiai Y, Ikeda M, Nishitani R et al. (2010) New approach for rice improvement using a pleiotropic QTL gene for lodging resistance and yield. Nat Commun 1:132

Ookawa T, Aoba R, Yamamoto T, Ueda T, Takai T, Fukuoka S, Ando T, Adachi S, Matsuoka M, Ebitani T et al. (2016) Precise estimation of genomic regions controlling lodging resistance using a set of reciprocal chromosome segment substitution lines in rice. Sci Rep 6:30572

Sato H, Matsumoto K, Ota C, Yamakawa T, Kihara J, Mizobuchi R (2015) Confirming a major QTL and finding additional loci responsible for field resistance to brown spot (Bipolaris oryzae) in rice. Breed Sci 65:170–175

Shim RA, Angeles ER, Ashikari M, Takashi T (2010) Development and evaluation of Oryza glaberrima Steud. chromosome segment substitution lines (CSSLs) in the background of O. sativa L. cv. Koshihikari. Breed Sci 60:613–619

Sugiura K, Honjo H, Hayashi M, Nonoyama T, Yamashita K, Torazawa A, Yamauchi A (2013) Research on the factors affecting kernel quality of rice cultivar ‘Koshihikari’ cultivated in Aichi Prefecture. Jpn J Crop Sci 82:262-269 (in Japanese with English summary)

Taguchi-Shiobara F, Ozaki H, Sato H, Maeda H, Kojima Y, Ebitani T, Yano M (2013) Mapping and validation of QTLs for rice sheath blight resistance. Breed Sci 63:301–308

Takai T, Nonoue Y, Yamamoto SI, Yamanouchi U, Matsubara K, Liang ZW, Lin HX, Ono N, Uga Y, Yano M (2007) Development of chromosome segment substitution lines derived from backcross between indica donor rice cultivar ‘Nona bokra’ and japonica recipient cultivar ‘Koshihikari’. Breed Sci 57:257–261

Takai T, Adachi S, Taguchi-Shiobara F, Sanoh-Arai Y, Iwasawa N, Yoshinaga S, Hirose S, Taniguchi Y, Yamanouchi U, Wu J et al. (2013) A natural variant of NAL1, selected in high-yield rice breeding programs, pleiotropically increases photosynthesis rate. Sci Rep 3:2149

Takai T, Ikka T, Kondo K, Nonoue Y, Ono N, Arai-Sanoh Y, Yoshinaga S, Nakano H, Yano M, Kondo M et al. (2014) Genetic mechanisms underlying yield potential in the rice high-yielding cultivar Takanari, based on reciprocal chromosome segment substitution lines. BMC Plant Biol 14:295

Takeuchi Y, Ebitani T, Yamamoto T, Sato H, Ohta H, Hirabayashi H, Kato H, Ando I, Nemoto H, Imbe T et al. (2006) Development of isogenic lines of rice cultivar Koshihikari with early and late heading by marker-assisted selection. Breed Sci 56:405–413

Tsunematsu H, Ando I, Nemoto H, Sunohara Y, Kato H, Hirabayashi H, Takeuchi Y, Maeda H, Sato H, Tanaka J et al. (2015) Breeding of "Koshihikari Kanto BL1", a Near Isogenic Line of "Koshihikari" with Blast Resistance Gene Pi9. Bull NARO Inst Crop Sci 15:77-93 (in Japanese with English summary)

Ujiie K, Kashiwagi T, Ishimaru K (2012) Identification and functional analysis of alleles for productivity in two sets of chromosome segment substitution lines of rice. Euphytica 187:325-337

Ujiie K, Ishimaru K (2013) Identification of chromosome regions affecting leaf area with rice chromosome segment substitution lines. Plant Prod Sci 16:31-36

Ujiie K, Yamamoto T, Yano M, Ishimaru K (2016) Genetic factors determining varietal differences in characters affecting yield between two rice (Oryza sativa L.) varieties, Koshihikari and IR64. Genetic Resources and Crop Evolution 63:97-123

Wang ZX, Sakaguchi S, Oka Y, Kitazawa N, Minobe Y (2005) Breeding of semi-dwarf Koshihikari by using genome breeding method. Breed Res 7 (Suppl 1.2):217 (in Japanese)

Yamaguchi T, Shinada H, Maeda H, Ebitani T (2010) Marker-assisted pyramiding of QTLs, the ‘Awa-akamai’ alleles increasing emergence of seedling in soil at low temperature, in the background of ‘Koshihikari’. Breed Res 12 (Suppl 2):239 (in Japanese)

Yamamoto T, Suzuki T, Suzuki K, Adachi S, Sun J, Yano M, Ookawa T, Hirasawa T (2016) Detection of QTL for exudation rate at ripening stage in rice and its contribution to hydraulic conductance. Plant Sci 242:270-277

Yamamoto T, Suzuki T, Suzuki K, Adachi S, Sun J, Yano M, Ookawa T, HirasawaT (2017) Characterization of a genomic region that maintains chlorophyll and nitrogen contents during ripening in a high-yielding stay-green rice cultivar. Field Crops Res 206:54-64

Yano K, Ookawa T, Aya K, Ochiai Y, Hirasawa T, Ebitani T, Takarada T, Yano M, Yamamoto T, Fukuoka S et al. (2015) Isolation of a novel lodging resistance QTL gene involved in strigolactone signaling and its pyramiding with a QTL gene involved in another mechanism. Mol Plant 8:303–314
